# In Response to “Concerns Regarding Masataka et al.'s ‘Revisiting the Gateway Drug Hypothesis for Cannabis’”

**DOI:** 10.1002/npr2.70093

**Published:** 2026-02-11

**Authors:** Yuji Masataka, Toshihiko Matsumoto

**Affiliations:** ^1^ Department of Neurosurgery St. Marianna University School of Medicine Kawasaki Japan; ^2^ General Incorporated Association Green Zone Japan Saitama Japan; ^3^ Department of Neurology Kumamoto Seijo Hospital Kumamoto Japan; ^4^ Department of Drug Dependence Research National Institute of Mental Health, National Center of Neurology and Psychiatry Tokyo Japan

## Abstract

We clarify Dr. Narita's concerns by replacing subjective wording with data‐based statements, emphasizing the descriptive—not causal—nature of our analysis, and noting that logistic regression was exploratory. Our findings show both progression and non‐progression pathways, indicating no single dominant gateway pattern among Japanese cannabis users.

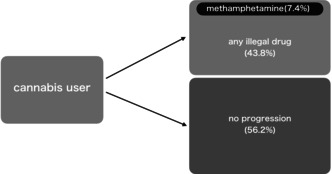

Dear Editor‐in‐Chief,

We thank Dr. Narita for the thoughtful comments on our article [1]. The critique centers on (1) our description of progression as “rare” vis‐à‐vis the reported odds, (2) the absence of a non‐user comparison group and the limits for causal inference, and (3) concerns about multivariable logistic regression (“Table 2 fallacy”). Below, we clarify each point.

## 1 On “Rare” and the Odds of Progression

Dr. Narita notes that our odds for transitioning to “any illegal drug” after cannabis were 0.78 (p ≈ 43.8%) and argues that describing progression as “rare” is misleading.

We appreciate this important observation and have revised our wording to avoid subjective phrasing. The sentence has been changed to the following empirically grounded statement: “Progression beyond cannabis was observed in approximately half of those who reported cannabis use as the third substance.” This revision provides a clear, data‐based description without invoking social or perceptual assumptions. We acknowledge that a 43.8% transition rate represents a nontrivial level of progression. Our intention was not to deny the possibility of progression, but rather to highlight that a substantial proportion (56.2%) of users did not proceed to any other illicit drugs. Taken together, the observed patterns indicate substantial variability in substance use trajectories following cannabis use. Accordingly, these findings do not support a uniformly applicable gateway sequence within this dataset.

## 2 On Comparison Groups and Causal Inference

We agree that causal inference requires appropriate comparators and design. Our aim was descriptive: to map sequences and conditional transitions among community cannabis users, not to estimate causal effects. We explicitly acknowledged limitations such as selection bias, recall bias, and generalizability. Our visualization also shows that alcohol and tobacco typically precede cannabis, and cannabis most often appears as the third substance—placing the putative “gateway” earlier in the sequence for Japan [2]. The higher lifetime exposure to some illicit drugs within our user sample (e.g., methamphetamine 10.4%) relative to the general population aligns with common‐liability and market‐structure explanations and does not constitute evidence of a necessary cannabis‐to‐other‐drug causal pathway; we discuss these alternatives in the paper. We concur that prospective cohorts with appropriate controls are needed to test causal claims.

## 3 On Multivariable Logistic Regression and the “Table 2 fallacy”

As a process clarification, the logistic regression analyses were not in our original submission; they were added at peer‐reviewers'request to provide supplementary, exploratory modeling. Their purpose was not causal estimation but to describe associations potentially informative for future hypothesis‐driven work. In the manuscript we framed these findings as associations (e.g., “appear to”), reported model fit (Hosmer–Lemeshow), and did not claim causal interpretation. We share the general concern highlighted by the Table 2 fallacy and agree that coefficients from convenience adjustments should not be over‐interpreted causally. In future studies, we plan to present explicit causal diagrams, differentiate confounders, mediators, and colliders, and, where appropriate, use target‐trial frameworks and g‐methods/sensitivity analyses.

## 4 Conclusion

In summary, we have clarified the three main points raised by Dr. Narita.

First, we have removed the subjective term “rare” and replaced it with a data‐based statement indicating that approximately half of cannabis users who reported cannabis as the third substance progressed to other drugs, while the other half did not. This balanced description reflects the coexistence of progression and non‐progression pathways among Japanese cannabis users.

Second, our study was descriptive in nature and did not aim to make causal inferences. We emphasized that appropriate comparison groups and prospective designs are necessary to establish causality, and that our findings align with alternative explanations such as common liability and market structure.

Third, the logistic regression analyses were exploratory and included at the request of peer reviewers. They were presented as associative—not causal—findings, with model fit reported to ensure transparency. We share the concern about the “Table 2 fallacy” and plan to incorporate formal causal frameworks and sensitivity analyses in future research.

Overall, our study contributes descriptive evidence that, in Japan, substance‐use trajectories involving cannabis are heterogeneous and not dominated by a single “gateway” pathway. We hope this clarification promotes a more nuanced and empirically grounded understanding of cannabis progression patterns within the Japanese context.

## Funding

The authors have nothing to report.

## Ethics Statement

The authors have nothing to report.

## Consent

The authors have nothing to report.

## Conflicts of Interest

The authors declare no conflicts of interest.

## Data Availability

Data sharing not applicable to this article as no datasets were generated or analyzed during the current study.
